# Genomic profiling of *Nitrospira* species reveals ecological success of comammox *Nitrospira*

**DOI:** 10.1186/s40168-022-01411-y

**Published:** 2022-11-30

**Authors:** Alejandro Palomo, Arnaud Dechesne, Anders G. Pedersen, Barth F. Smets

**Affiliations:** 1 Department of Environmental and Resource Engineering, Technical University of Denmark, Kgs Lyngby, Denmark; 2 School of Environmental Science and Engineering, Southern University of Science and Technology, Shenzhen, China; 3 Section for Bioinformatics, Department of Health Technology, Technical University of Denmark, Copenhagen, Denmark

## Abstract

**Background:**

The discovery of microorganisms capable of complete ammonia oxidation to nitrate (comammox) has prompted a paradigm shift in our understanding of nitrification, an essential process in N cycling, hitherto considered to require both ammonia oxidizing and nitrite oxidizing microorganisms. This intriguing metabolism is unique to the genus *Nitrospira*, a diverse taxon previously known to only contain canonical nitrite oxidizers. Comammox *Nitrospira* have been detected in diverse environments; however, a global view of the distribution, abundance, and diversity of *Nitrospira* species is still incomplete.

**Results:**

In this study, we retrieved 55 metagenome-assembled *Nitrospira* genomes (MAGs) from newly obtained and publicly available metagenomes. Combined with publicly available MAGs, this constitutes the largest *Nitrospira* genome database to date with 205 MAGs, representing 132 putative species, most without cultivated representatives. Mapping of metagenomic sequencing reads from various environments against this database enabled an analysis of the distribution and habitat preferences of *Nitrospira* species. Comammox *Nitrospira*’s ecological success is evident as they outnumber and present higher species-level richness than canonical *Nitrospira* in all environments examined, except for marine and wastewaters samples. The type of environment governs *Nitrospira* species distribution, without large-scale biogeographical signal. We found that closely related *Nitrospira* species tend to occupy the same habitats, and that this phylogenetic signal in habitat preference is stronger for canonical *Nitrospira* species*.* Comammox *Nitrospira* eco-evolutionary history is more complex, with subclades achieving rapid niche divergence via horizontal transfer of genes, including the gene encoding hydroxylamine oxidoreductase, a key enzyme in nitrification.

**Conclusions:**

Our study expands the genomic inventory of the *Nitrospira* genus, exposes the ecological success of complete ammonia oxidizers within a wide range of habitats, identifies the habitat preferences of (sub)lineages of canonical and comammox *Nitrospira* species, and proposes that horizontal transfer of genes involved in nitrification is linked to niche separation within a sublineage of comammox *Nitrospira.*

Video Abstract

**Supplementary Information:**

The online version contains supplementary material available at 10.1186/s40168-022-01411-y.

## Background

The biological oxidation of ammonia to nitrate via nitrite, termed nitrification, is an essential process in terrestrial and aquatic environments. Nitrification links oxidized and reduced pools of inorganic nitrogen, contributes to nitrogen loss from agricultural soils reducing fertilization efficiency [1], is a crucial process in water quality engineering [2], and can lead to the production of nitrous oxide, a strong greenhouse gas [3]. For more than one century, this central process in the nitrogen cycle was assumed to be a two-step process catalyzed by two distinct functional groups: ammonia-oxidizing microorganisms (AOM–consisting of bacteria and archaea) and nitrite-oxidizing bacteria (NOB). In 2015, microorganisms capable of the complete oxidation of ammonia to nitrate (complete ammonia oxidation *aka* comammox) were discovered [4, 5] within the *Nitrospira* genus. This genus was previously considered to only harbor nitrite oxidizers (here also referred to as canonical *Nitrospira*) capable of using alternative metabolisms such as hydrogen or formate oxidation, but not ammonia oxidation [6].

Phylogenetic evidence indicates a complex evolutionary history of comammox capability and it is currently unclear whether or not it was present in ancestral *Nitrospira* [7]. Clearly, the gain (or the loss) of such a trait must have had strong ecological consequences. Indeed, comammox *Nitrospira* can capture a much greater amount of energy from ammonia oxidation than canonical *Nitrospira* from nitrite oxidation, but most likely at the cost of a lower maximum growth rate [8, 9]. In addition, comammox and canonical *Nitrospira* directly compete with different guilds of nitrifiers (AOM and NOB, respectively), resulting in a very different selective landscape. Yet, we have little understanding of how these markedly different metabolic strategies affect *Nitrospira*’s current ecological distribution and how this distribution relates to its evolutionary history.

Phylogenetic diversity within *Nitrospira* is high [6]: the genus consists of at least six lineages with pronounced divergence at the 16S rRNA level (sequence similarities < 90%). Its members are environmentally widespread [6], and are found in soils [10], freshwaters [11], oceans [12], groundwaters [13], and technical systems [14, 15]. Comammox *Nitrospira* species have been detected in different natural environments such as soils, rivers, lakes or coastal wetlands [16–19]; as well as in engineered systems such as waste and drinking water treatment plants [4, 20]. Estimates from a limited number of sites indicate that comammox *Nitrospira* is abundant in drinking water treatment plants (DWTP) [21], and to a lesser extent, in wastewater treatment plants (WWTP) [22] and soils [23, 24]. So far, the distribution or abundance estimates of comammox *Nitrospira* have been based on either functional gene (subunit A of the ammonia monooxygenase (*amoA*)) amplicon-based profiling [16, 25, 26] or on limited genome-based profiles in specific environments [23, 27–29]. As a consequence, our view of the distribution, abundance, and diversity of *Nitrospira* is fragmented and likely incomplete. Shotgun metagenomic sequencing profiling has been successfully used to disclose the ecological patterns of various microbial populations at the large scale [30–32]. In this study, we used metagenome assembled genomes (MAGs) to create the largest *Nitrospira* genomes database to date. By comparing the raw sequencing reads in metagenomic data to these genomes (“read recruitment”), we determined the presence and abundance of *Nitrospira* species in various environments across the globe and elucidated how environment and spatial distance affect *Nitrospira* distribution, and to what degree *Nitrospira* phylogeny associates with its ecology. Our analysis provides a global survey of *Nitrospira* distribution and abundance with species-level resolution and unravels the niche preferences of the different comammox *Nitrospira* types.

## Results

### Genomic characteristics of *Nitrospira* genomes

**Fig. 1:**
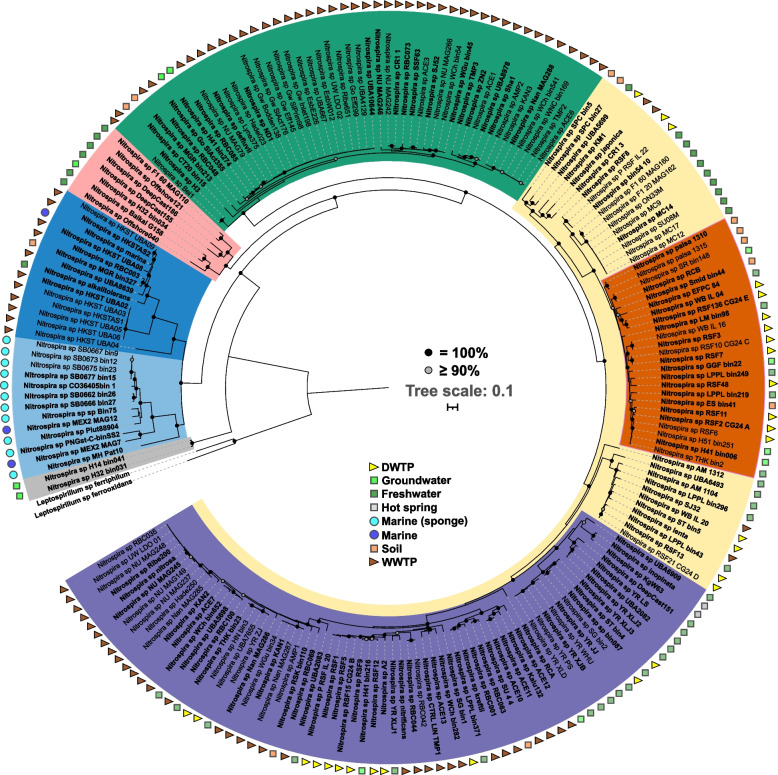
Phylogenomic affiliation of 205 *Nitrospira* MAGs inferred from the concatenation of 120 amino acid sequences. Lineages and sublineages are shown with colors: lineage I, green; lineage II, yellow (comammox clade A, purple; comammox clade B, brown); lineage IV, blue (dark colour, type A; light color, type B); other lineages, grey and pink. *Leptospirillum* was used to root the tree. Representative of putative *Nitrospira* species (ANI ≥ 96% are considered members of the same species) are highlighted in boldface. The strength of support for internal nodes as assessed by bootstrap replicates is indicated as colored circles. Symbols next to the MAGs names denote the environment from where the MAGs were recovered

Using a combination of multiple automatic binning tools followed by several refinement steps to improve the bin quality, we retrieved 55 metagenome-assembled *Nitrospira* genomes (MAGs) from newly obtained and publicly available metagenomes (Table S1). In addition, we downloaded *Nitrospira* MAGs from public databases (up to January 2022), resulting in a database of 205 MAGs from drinking water treatment plants (DWTP) (*n* = 29), freshwater (*n* = 45), groundwater (*n* = 10), marine (*n* = 14), soil (*n* = 11), wastewater treatment plants (WWTP) (*n* = 92) and other environments (*n* = 4); from across the globe (Canada, *n* = 12; China, *n* = 58; Europe, *n* = 61; Oceania, *n* = 12; USA, *n* = 37; others, *n* = 25) (Table S2). The phylogenomic analysis of the 205 MAGs placed 48 MAGs into *Nitrospira* lineage I, 121 into lineage II, 27 into lineage IV, and 9 into other lineages (Fig. 1). Two genomes recovered from groundwater samples (*Nitrospira* sp. H14_bin041 and *Nitrospira* sp. H32_bin031) not associated to known lineages and phylogenetically distant to other nitrifying-*Nitrospira* spp. were included in the catalog as they both contain genes involved in nitrite oxidation (*nxrA* and *nxrB*). The 205 MAGs span 132 putative species (further on simply referred to as ‘species’) using a threshold average nucleotide identity (ANI) of ≥ 96% (Figure S1 and Table S2). Of the 205 MAGs, 93 are comammox *Nitrospira* (69 clade A and 24 clade B) (Fig. 1 and Table S1). Similarly, out of the 132 *Nitrospira* species, 66 are comammox *Nitrospira* (49 clade A and 17 clade B) (Fig. 1). Average genome completeness and contamination were estimated at 89% (70 to 98%) and 1% (0 to 5%), respectively (Table S2). *Nitrospira* MAGs have an average genome size of 3,435,709 ± 860,289 bp, and contain 3541 ± 844 genes. Although there was no significant difference in the genome completeness across different *Nitrospira* lineages, there were obvious dissimilarities in the genome size and gene number (Table 1). The variability in genome size is marked within Lineage IV, with one branch (IV type A), associated with MAGs from wastewater treatment plants, characterized by genomes much larger than those ones in the second branch, dominated by MAGs from marine/sponges (IV type B). Thus, genomes in the lineage IV type B and genomes belonging to two lineages distinct from lineages I, II, and IV (referred to as ‘other lineages’) are significantly smaller and harbor less genes than other *Nitrospira* genomes (*p* < 0.05). The GC content of the genomes is 56.2% ± 2.8. The lowest GC content was detected in the lineage IV type A genomes (49.8% ± 0.9), while lineage I (58.8% ± 0.6) and genomes assigned to other lineages have highest GC (62.0% ± 1.3) (Table 1). Interestingly, the two clearly separated groups within Lineage IV (A and B) are significantly different in genome size, number of genes and GC content (Table 1 and Fig. 1).

**Table 1:** Genomic characteristics of the *Nitrospira* MAGs. Differences between the mean of each feature across the different (sub)lineages were assessed by a Dunn’s test. For each feature, values with the same letter have means not significantly different from each other (*p* < 0.05)

	Genomes	Completeness (%)	Size (Mbp)	Genes	GC (%)
Lineage I	48	91.0 ± 6.6 ^a^	3.9 ± 0.5 ^b^	3919 ± 404 ^a^	58.8 ± 0.6 ^c^
Lineage II (comammox clade A)	69	88.8 ± 8.2 ^a^	3.6 ± 0.6 ^ab^	3810 ± 557 ^a^	55.5 ± 1.1 ^a^
Lineage II (comammox clade B)	24	89.0 ± 6.1 ^a^	3.4 ± 0.6 ^ac^	3594 ± 719 ^a^	56.0 ± 0.7 ^ab^
Lineage II (*lenta* type)	10	88.3 ± 6.4 ^a^	3.3 ± 0.5 ^abcd^	3400 ± 354 ^ab^	58.1 ± 0.5 ^bcd^
Lineage II (others)	18	89.2 ± 6.3 ^a^	2.4 ± 0.9 ^cd^	2553 ± 870 ^b^	58.2 ± 1.5 ^cd^
Lineage IV type A	14	87.5 ± 8.8 ^a^	4.1 ± 0.9 ^ab^	4227 ± 1030 ^a^	49.8 ± 0.9 ^e^
Lineage IV type B	13	92.6 ± 4.9 ^a^	2.4 ± 0.3 ^d^	2359 ± 334 ^b^	56.2 ± 2.9 ^abd^
Other lineages	9	84.2 ± 9.7 ^a^	1.9 ± 0.6 ^d^	2095 ± 525 ^b^	62.0 ± 1.3 ^c^

### Comparison of *Nitrospira* genomes

A pangenomic analysis of 205 *Nitrospira* MAGs grouped a total of 723,029 coding sequences (CDS) into 21,450 gene clusters (GCs), with a *Nitrospira* core genome containing 1190 GCs (Table S3), a similar number and metabolic content to our previous study of 16 *Nitrospira* genomes [7] (59,744 CDS grouped into 12,337 GCs, with a core genome consisting of 1382 GCs). The core genome includes genes for the nitrite oxidation pathway, the reductive tricarboxylic acid cycle for CO_2_ fixation (rTCA), gluconeogenesis, the pentose phosphate pathway, and the oxidative TCA cycle. Chlorite dismutase and copper-containing nitrite reductase (*nirK*) are also present in the core genome. We identified a total of 49 comammox-specific GCs; 18 and 3 of these GCs have highest sequence similarity to homologs in betaproteobacterial ammonia-oxidizing bacteria (AOB) and gammaproteobacterial methane oxidizers, respectively (Table S3). These genes encode the ammonia oxidation pathway, as well as specific urea transporters and copper homeostasis proteins (Table S3). We identified 47 and 25 comammox clade A and clade B-specific GCs, respectively, mostly with unknown function (Table S3). In addition, each *Nitrospira* genome harbors an average of 161 unique gene clusters, most of unknown function.

### Comammox *Nitrospira* spp. are widely distributed and often dominant over canonical *Nitrospira*

**Fig. 2:**
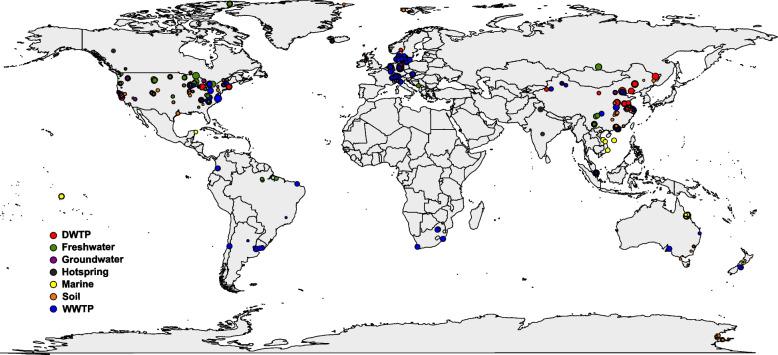
Location of the 598 metagenomes where *Nitrospira* species were detected. The samples represent 5 distinct environments including DWTP (*n* = 40), freshwater (*n* = 127), groundwater (*n* = 63), hot spring (*n* = 13), marine (*n* = 27), soil (*n* = 180), WWTP (*n* = 147). The size of the circle denotes abundance of the *Nitrospira* species in the metagenomes (log_100_ of RPM)

We characterized the distribution of the 132 *Nitrospira* species in 788 metagenomes across eight broadly defined environments (drinking water treatment plant (DWTP), *n* = 40; freshwater, *n* = 167; groundwater, *n* = 86; hot spring, *n* = 36; marine, *n* = 75; soil, *n* = 206; wastewater treatment plant (WWTP), *n* = 177; others, *n* = 1) across the globe (China, *n* = 139; Europe, *n* = 128; USA, *n* = 309; others, *n* = 212) (Table S4). *Nitrospira* species were detected in 598 metagenomes (Fig. 2). The most widely distributed *Nitrospira* species in the investigated metagenomes were the canonical lineage I *Nitrospira* sp. ND1 (frequency of occurrence of 18%) and *Nitrospira* sp. UBA10644 (16%), and the clade B comammox *Nitrospira* sp. LM_bin98 (16%) (Figure S2). Forty-one species were found in less than 1% of the metagenomes, including several of the *Nitrospira* species with a representative isolate or enrichment such as *Nitrospira* sp. KM1, *N. japonica*, *N. marina* (Figure S2).

**Fig. 3:**
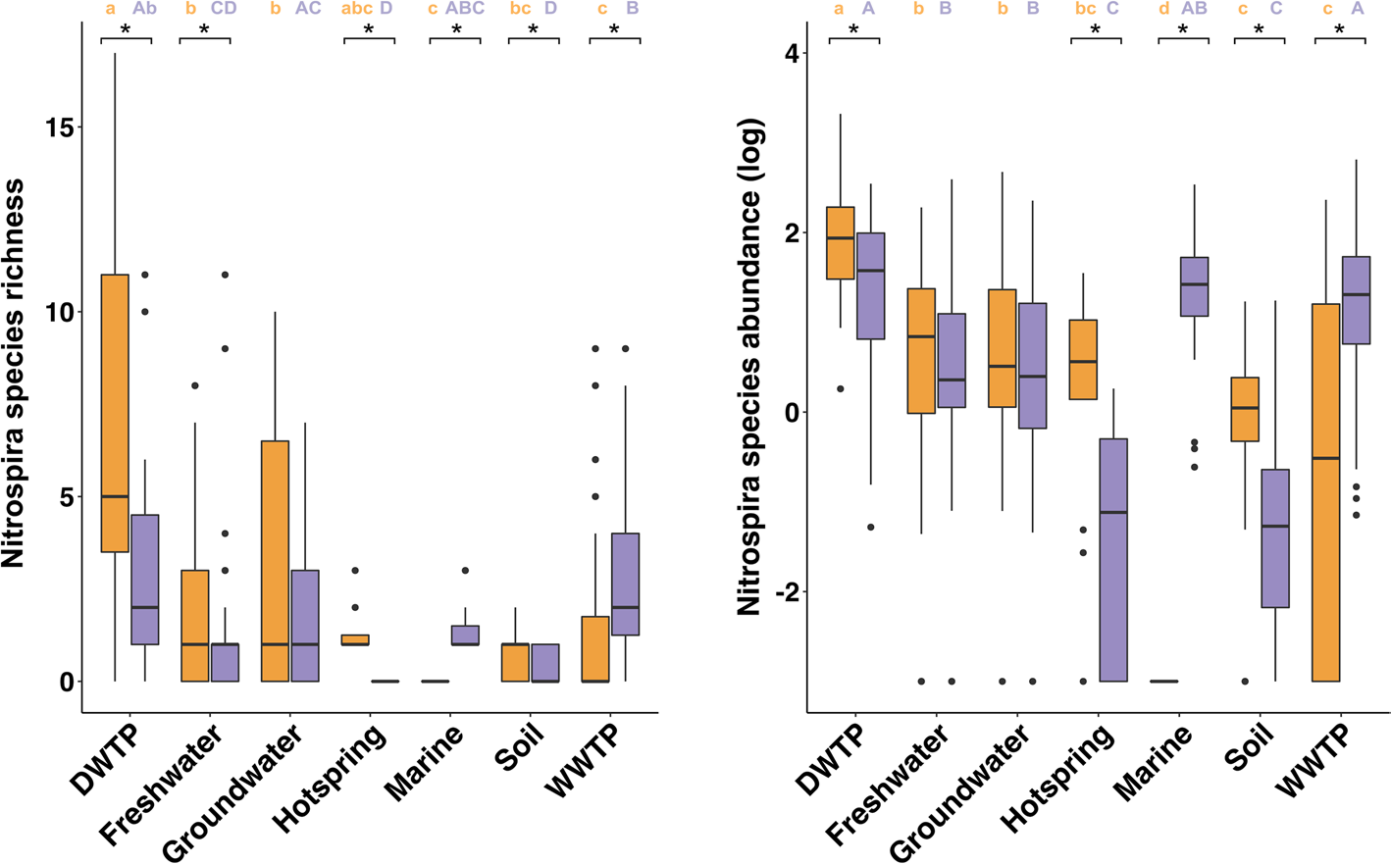
**A** Richness and **B** abundance (log_10_ of RPM) of comammox (orange) and canonical (purple) *Nitrospira* species across habitats. Differences between the mean of the richness or abundance of comammox or canonical *Nitrospira* across habitats were assessed by a Dunn’s test; habitats with the same letter have means not significantly different from each other (*p* < 0.05), with small letter for comammox *Nitrospira* and capital letters for canonical *Nitrospira* species. Asterisk (*) indicates significant difference (*P* < 0.05) between the mean richness or abundance of comammox and canonical *Nitrospira* in a specific habitat. Boxes represent the first quartile, median, and third quartile of distribution values, and whiskers of × 1.5 interquartile range

All the DWTP (40/40), most of the WWTP (147/177), groundwater (63/86), freshwater (127/167), and soil metagenomes (180/206); and about one third of the hot spring (13/36) and marine metagenomes (27/75) have at least one *Nitrospira* species. The *Nitrospira* species richness per metagenome is highest in the DWTP metagenomes (10.0 ± 6.7), followed by groundwater (5.3 ± 5.4), WWTP (4.1 ± 3.5) and freshwater (2.9 ± 2.9), while it is lowest in the hot spring (1.4 ± 0.7), marine (1.4 ± 0.7) and soil metagenomes (1.1 ± 0.4). The average number of detected comammox *Nitrospira* species exceeds that of non-comammox species in DWTP, freshwater, and hot springs, while the opposite was observed for WWTP and marine samples (*P* < 0.05), where comammox was not detected (Fig. 3A). No significant difference was found in groundwater metagenomes (Fig. 3A). The DWTP metagenomes have the highest abundance of *Nitrospira* spp., while hot spring and soil metagenomes have the lowest abundance of *Nitrospira* MAGs (Fig. 3B). Comammox *Nitrospira* constitute the majority of *Nitrospira* species in DWTP, hot spring, and soil metagenomes, while canonical species are significantly more abundant than comammox *Nitrospira* in marine and WWTP samples (*P* < 0.05) (Fig. 3B).

### Environment determines *Nitrospira* spp. distribution, without large-scale biogeographical signal

**Fig. 4:**
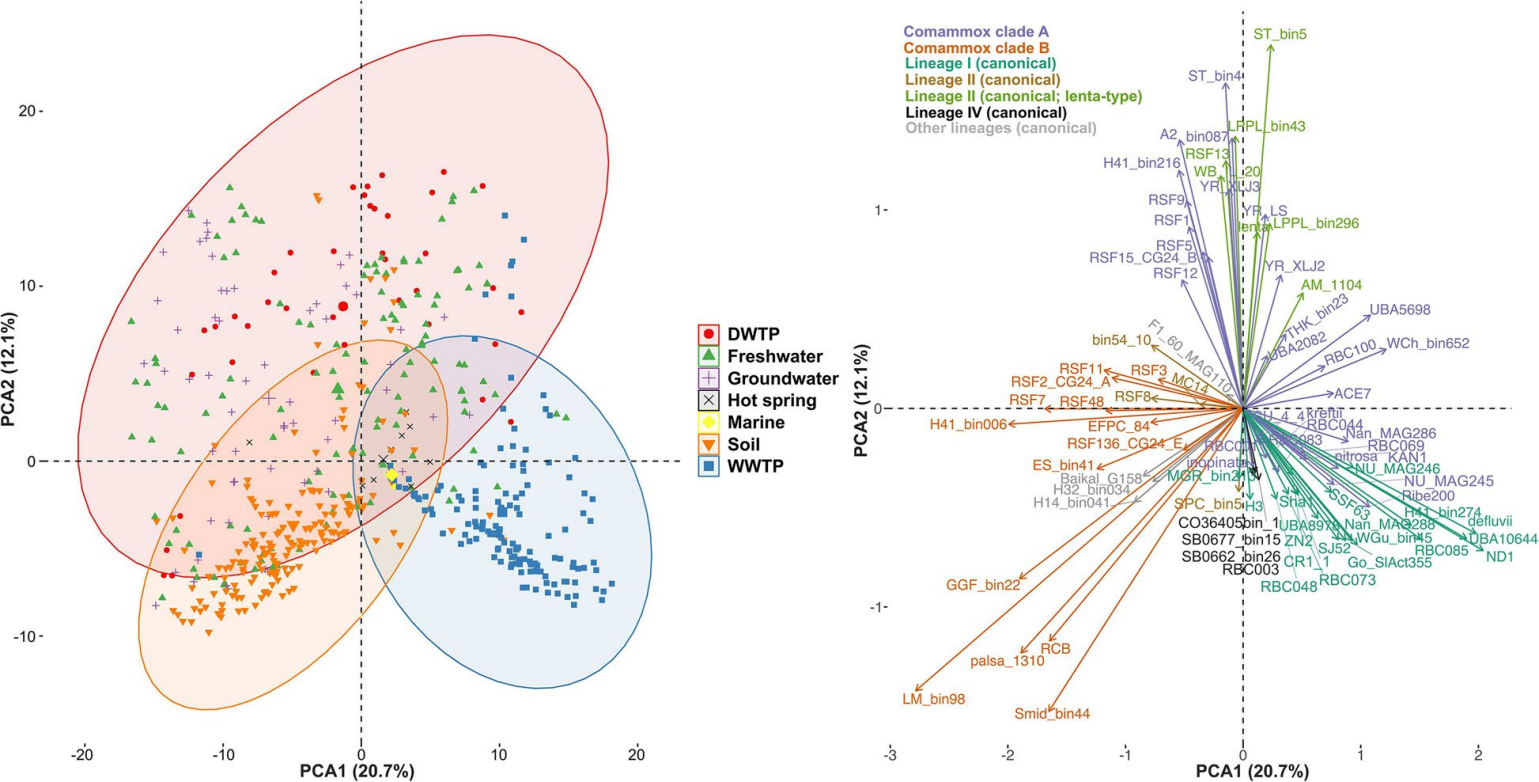
Principal component analysis based on the centered-log transformed abundance of *Nitrospira* species across 598 metagenomes. Left: plot of the metagenomes; 95% confidence ellipses are shown. Right: plot of the *Nitrospira* species. The contributions of PC1 (horizontal axis) and PC2 (vertical axis) are 21% and 12%, respectively

To examine the distribution pattern of *Nitrospira* species, we conducted a principal component analysis (PCA) of the relative abundance of *Nitrospira* species in the metagenomes. WWTP metagenomes clearly cluster and separate from DWTP metagenomes (Fig. 4A); soil metagenomes also cluster together, while groundwater and freshwater metagenomes have more variable *Nitrospira* compositions (Fig. 4A). Within each habitat, we found, in most cases, a weak, but significant correlation between geographical distance and *Nitrospira* community dissimilarity (Figure S3). However, these correlations nearly disappear when samples within small distances were excluded from the analysis, with the exception of DWTP (Figure S3).

Analysis of the *Nitrospira* species abundances across the metagenomes showed that *Nitrospira* spp. belonging to the same (sub)lineages tend to co-occur, and also, revealed the existence of niche separation between *Nitrospira* (sub)lineages (Fig. 4B, Figure S4 and Figure S5). Lineage I species are primarily found in WWTP metagenomes while the distribution of lineage II species is more varied. Clade A comammox *Nitrospira* species are distributed in two clusters (Fig. 4B, Figure S4). Some of the clade A species co-occur with the *Nitrospira* lineage I species in WWTP samples, while the other clade A species are more typical of DWTP along with some of the groundwater and freshwater metagenomes, and co-occur with the lineage II *Nitrospira lenta*-type species. The clade B comammox *Nitrospira* species are frequently present in DWTP, groundwater, freshwater, and soil metagenomes (Fig. 4B and Figure S4). Lineage IV species are characteristic of marine samples, while *Nitrospira inopinata* is the dominant species present in hot springs (Fig. 4B and Figure S4).

### Relation between ecological and phylogenetic similarities

We assessed which feature better explained habitat preference by comparing, for pairs of species, similarity in their environmental distribution (as measured by the correlation of their abundance across samples) with overall genomic similarity (as measured by the average amino acid identity between their genomes). Similarity in environmental distribution was also compared to sequence similarity of the key nitrification enzymes ammonia monooxygenase (AMO), hydroxylamine dehydrogenase (HAO) and nitrite oxidoreductase (NXR). We found that genomic similarity between pairs of species is positively correlated to similarity in habitat preference (Mantel statistic *r* = 0.47, *P* < 0.001) (Table 2). A similar correlation was obtained when only comammox species or canonical species were evaluated (*r* = 0.63, *P* < 0.001; *r* = 0.69, *P* < 0.001). This correlation was especially strong when only the canonical *Nitrospira* species from lineage I and II were considered (*r* = 0.83, *P* < 0.001). Similar trends as for AAI were observed when using a set of 120 translated single-copy genes, although with slightly weaker correlation values (Table 2). Regarding the enzymes involved in nitrification, we observed very week correlations between NxrB similarity, considered a powerful functional and phylogenetic marker [6], and habitat preference with the exception of canonical *Nitrospira* species affiliated to lineage I and II (*r* = 0.70, *P* < 0.001). The correlation between AmoA sequence similarity and habitat similarity was weaker than for whole genome similarity for comammox *Nitrospira* species (*r* = 0.46, *P* < 0.001). HaoA similarity, however, displayed a stronger correlation with habitat similarity (*r* = 0.60, *P* < 0.001).

**Table 2:** Relationship (Mantel statistic *r*) between habitat similarity and genetic similarity between *Nitrospira* species pairs for different markers

	Whole genome	Single-copy genes	AmoA	HaoA	NxrB
*Nitrospira*	0.47***	0.34***	–	–	0.08*
Comammox	0.69***	0.57***	0.46***	0.60***	0.23**
Canonical	0.63***	0.52***	–	–	0.10
Canonical (Lineage I and II)	0.83***	0.71***	–	–	0.70***

*p < 0.05; **p < 0.01; ***p < 0.001

### Hydroxylamine dehydrogenase is potentially involved in recent niche separation within clade A

**Fig. 5:**
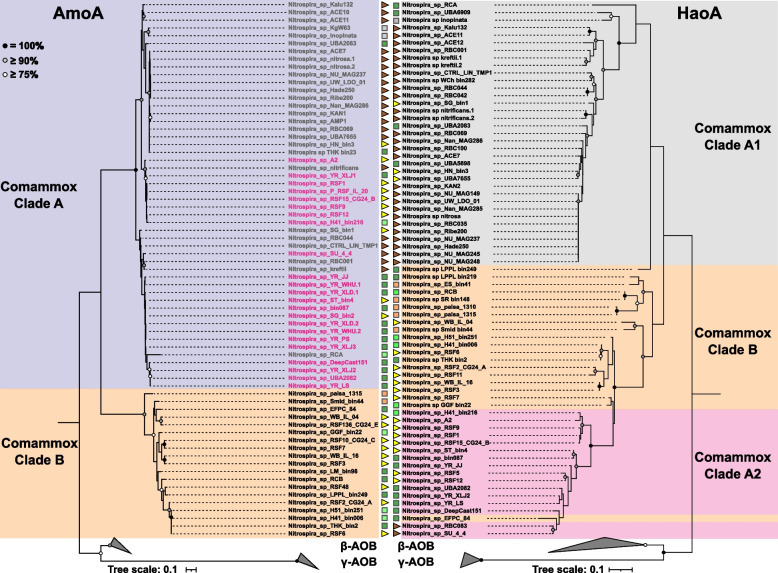
Maximum likelihood phylogenetic trees of *Nitrospira* spp. based on (left) AmoA and (right) HaoA amino acid sequences. Only complete or nearly complete sequences are included. The strength of support for internal nodes was assessed by performing bootstrap replicates, with the obtained values shown as colored circles (top left legend). The comammox *Nitrospira* clades and subclades are indicated by colored boxes, and the colour font of clade A sequences in the AmoA tree refers to their membership on the HaoA tree. Gamma- and betaproteobacterial AOB sequences were used to root the trees. Shapes next to the sequence names denote the environment from where the MAGs were recovered (color code as in Fig. 1)

These results indicate that the hydroxylamine dehydrogenase might be a better indicator than other nitrification enzymes of niche preference for comammox *Nitrospira*. Consistent with this, phylogenetic analysis of HaoA showed a division of clade A into two subclusters (referred to as clade A1 and clade A2, respectively) similar to the one identified from habitat preference patterns (Fig. 4 and Figure S4). Indeed, clade A2 sequences are clearly separated from those of clade A1, and more closely related to some of the clade B species (Fig. 5). This contrasts with the clear monophyletic separation of the two comammox clades (A and B) based on phylogeny of AmoA (Fig. 5), which is also supported by the phylogenetic analyses of the concatenation of 120 translated single-copy genes (Fig. 1). Further, we detected two *nirK* genes (encoding a copper-containing nitrite reductase) next to the HAO cluster in the clade A2 genomes. This gene synteny was also found in several of the clade B genomes, but never in the clade A1 genomes (Figure S6). This shared synteny of the HAO genetic region suggests a horizontal gene transfer event between clade A2 and clade B (as also proposed in our earlier evolutionary analysis [7]) instead of convergent evolution of the hydroxylamine dehydrogenase.

## Discussion

This study represents the first effort to analyze the global distribution and ecological niches of *Nitrospira* species, including comammox *Nitrospira*. We combined a large number of *Nitrospira* MAGs retrieved in this study with publicly available genomes to build a dataset consisting of 205 medium to high quality *Nitrospira* genomes (> 70% completeness and < 5% contamination). These genomes were recovered from very different environments and geographical areas, and spanned five different lineages. This dataset properly covers the most ubiquitous and species-rich lineages (I and II) as well as both comammox clades. It also provides a unique insight into the phylogenomics of lineage IV which demonstrates that this deep-branching lineage experienced stronger genomic changes than the rest of the lineages and should probably be considered as comprising of two separate lineages, as previously proposed from 16S rRNA gene sequence analysis [33]), with starkly contrasting genomic features (genome size, gene number, and GC content). In addition, genomes from other lineages lacking cultivated representatives were present in the database. On the other hand, no MAGs from the previously established lineages V and VI were represented in the database. Nevertheless, only one enrichment (*Candidatus* Nitrospira bockiana; lineage V), one isolate (*Nitrospira calida*; lineage VI) and few 16S rRNA sequences have been recovered from these lineages that are believed to occupy very specific habitats [6, 34, 35]. Overall, the database built in this study represents the larger genomic dataset used to study the genomic characteristics and ecology of the *Nitrospira* genus.

We quantified the relative abundance and distribution of *Nitrospira* species in different habitats by exploiting large amounts of shotgun metagenomic sequencing data. Thus, we mapped metagenomic sequencing reads against a *Nitrospira* database of 15 universal single-copy genes from the *Nitrospira* species retrieved in our study [31]. The uncovered diversity of *Nitrospira* species, and specifically the vast diversity within lineage II *Nitrospira*, reflects the species-level diversity previously estimated from 16S rRNA sequences [6]. Our analysis confirms the previously described ubiquity of *Nitrospira* [6] and reveals the extent of the ecological success of comammox *Nitrospira*. We detected comammox species within a wide range of climatic zones, from polar (soils in Svalbard and Antarctica) to tropical (soils in Vietnam, and fresh and wastewater in Singapore) and temperate zones. We found that comammox *Nitrospira* coexist with and are more abundant than canonical *Nitrospira* in all studied habitats except in WWTP and marine samples. This hitherto unrecognized dominance of comammox *Nitrospira* implies that, until recently, by equating detection of *Nitrospira* 16S rRNA or *nxrB* with strict NOB metabolism, the number of ammonia oxidizers in the environment has been systematically and significantly underestimated. Among the studied habitats, comammox *Nitrospira* is especially diverse and abundant in drinking water treatment systems, which are characterized by surface-attached microbial communities and low ammonium fluxes [15, 36], a suitable environment for high growth yield microorganisms such as comammox *Nitrospira* [8, 37]. On the other hand, canonical *Nitrospira* clearly outnumber comammox *Nitrospira* in WWTP. These results are line with a study conducted on samples collected from hundreds of WWTP across the world, which shows canonical *Nitrospira* lineage I species as most abundant *Nitrospira* [38]. This predominance of canonical nitrite oxidizers in WWTP is consistent with the proposition that nitrogen-rich environments select for division of labour because it maximizes growth rates [8, 39]. However, in some full-scale WWTP [22, 40] and lab-scale reactors [41], comammox *Nitrospira* have been found more abundant than canonical ammonia oxidizers, suggesting that factors beyond nitrogen fluxes influence comammox *Nitrospira* fitness in these systems. Based on a survey of 14 WWTP, Cotto et al. (2020) concluded that long solids retention time favor the prevalence of comammox bacteria [42]. *Nitrospira* MAGs were detected in marine metagenomes, although in most cases, they were samples collected from coral reef or/and sponges. This is consistent with *Nitrospira* being a minor nitrite oxidizing bacterium in oceans [6]. Comammox were not detected in the marine metagenomes, as all *Nitrospira* species identified in this habitat were restricted to lineage IV. Previously studies have detected comammox in estuaries [43, 44] and coastal sediments [45], but consistently with our findings, no complete ammonia oxidizers have been found in open ocean water. Multiple studies have highlighted the relevance of comammox *Nitrospira* spp. in different soil systems [19, 46–49], however, our analysis showed relatively low presence, abundance and richness of *Nitrospira* spp. in soil metagenomes. This habitat generally contains a large diversity of microorganisms, and recovery of MAGs from this kind of complex metagenomes can be challenging. Therefore, it might be possible that *Nitrospira* species from soils are underrepresented in our dataset. Based on *amoA* amplicon sequencing a variety of comammox *Nitrospira*, especially assigned to clade A, have been detected in soils [19, 47, 50]. This contrasts with the limited number of comammox *Nitrospira* clade A MAGs recovered from soils metagenomes in our catalog. To circumvent this potential underrepresentation, we extended our analysis beyond the species level (ANI ≥ 95%) by mapping metagenomics reads to our database using an ANI cutoff of 75% (genus level). The presence and number of *Nitrospira* spp. detected in the soil metagenomes increased, however, richness and abundance were still lower than in most other environments (Table S5). Nevertheless, efforts to recover more genomes from soil environments or conducting global-scale studies using others approaches (such as qPCR) would help to confirm our observations.

In contrast to what has been observed for other bacterial taxa such as *Streptomyces* [51], SAR11 [52] or Acidobacteria [53], we did not detect a strong biogeographical signal for *Nitrospira* spp., with the exception of drinking water treatment plants and, to a lesser extent, wastewater treatment plants, where dissimilarity of the *Nitrospira* communities correlates with geographical distance. However, due to the limited number of DWTP samples, a larger dataset is needed to confirm this observation. For all other habitats, the correlation nearly disappears after few tens of kilometers, which might correspond to the limit of effective dispersal of *Nitrospira* species or might reflect the scale of variability of environmental parameters that affect *Nitrospira* spp. Environmental filtering controls *Nitrospira* species distribution and, since closely related species tend to share habitats preference, it results in a clear phylogenetic clustering of *Nitrospira* types in environmental communities. As observed for other microbial taxa [54, 55], dissimilarity in habitat preference generally increases with genomic dissimilarity, with the strongest correlation for canonical *Nitrospira* species. This pattern is consistent with progressive phylogenetic and ecological divergences, as expected from genetic drift or from random fluctuation in natural selection [56]. Yet, this pattern of congruency between genetic similarity and habitat similarity is not always true when considering the genes essential to nitrification. Indeed, the similarity in NxrB sequences, used as functional and phylogenetic markers for nitrite oxidation [10], does not predict habitat similarity for comammox *Nitrospira*. In contrast, HaoA sequence similarity strongly correlates with comammox *Nitrospira* habitat preference. This is because HaoA phylogeny matches the striking niche separation we observed within clade A comammox *Nitrospira* species. While clade A2 species were mostly found in DWTP, groundwater and freshwater sharing habitats with clade B comammox species, comammox clade A1 species primarily occupy the habitat typical of lineage I *Nitrospira* species (WWTP). This niche separation is not captured by *amoA* phylogeny and, as result, the distinction of two subclades of clade A comammox *amoA* (also named A1 and A2 [25]), provides less accurate information on habitat preference. This can be illustrated by the fact that *amoA*-based clade A1 has both representatives that frequently occur in WWTP (*N. nitrosa* cluster) and others (*Nitrospira* sp. RFS1 cluster) that are typical of DWTP, groundwater and freshwater samples (Fig. 4 and Figure S4).

Ecological divergence of closely related types can emerge from gene acquisition through horizontal gene transfer [57–59] or by changes in existing genes whose divergence can be accelerated by natural selection [60–62]. The high sequence divergence for HAO subunits between clade A1 and clade A2 genomes, and the similarity between clade A2 HAO and clade B HAO is unlikely to result from convergent evolution, and horizontal gene transfer is thus the likely cause of the ecological divergence between clade A1 and clade A2 comammox *Nitrospira*. Indeed, clade A2 and some of the clade B genomes share a unique *hao* synteny that includes a duplicate *nirK*, which is absent in clade A1 genomes. Such transfer event between clade A2 and B had been proposed in a previous, more limited evolutionary analysis [7]. It is plausible that this transfer would provoke a niche modification in the recipient as HAO is one of the key enzymes for ammonia oxidation [3]. The apparent co-transfer of adjacent *nirK* genes is intriguing, as NirK has recently been posited as essential in ammonia oxidation [63]. Nevertheless, although our analysis shows that the genomes involved in this proposed horizontal gene transfer tend to share similar habitats, further ecophysiological analyses are needed to confirm the basis of the ecological divergence within clade A. This will require continuous efforts at isolating representatives of all comammox *Nitrospira* (sub)clades, beyond the few currently isolate and enrichments, which belong to comammox clade A1 [5, 37, 64].

## Conclusions

This study represents the most extensive survey of the *Nitrospira* genus at genome level performed to date. Complete ammonia oxidizers are present in a large variety of habitats, biomes and global regions, and often dominate over canonical *Nitrospira*. Our analysis suggests that the type of environment governs *Nitrospira* species distribution, without large-scale biogeographical signal. We identified the habitat preferences of (sub)lineages of canonical and comammox *Nitrospira* species, and proposed that horizontal transfer of genes involved in nitrification could be linked to niche separation within a sublineage of comammox *Nitrospira.* Together, these findings provide deeper insights into the ecology of an important player of the biogeochemical nitrogen cycle. Future studies involving canonical ammonia oxidizers are needed to determining the functional redundancy and niche differentiation of comammox, ammonia oxidizing bacteria and ammonia oxidizing archaea at global scale.

## Methods

### Sample collection and DNA extraction

Filter material (15 ml) was collected from 2 locations or from 1 location at two different times at the top of the filters of 12 Danish waterworks using a 1% hypochlorite-wiped stainless-steel grab sampler. Filter material was immediately placed into cryotubes, immersed in liquid nitrogen and stored at – 80 °C for further analysis. DNA was extracted from 0.5 g of sand material using the MP FastDNA Spin Kit (MP Biomedicals LLC, Solon, USA) as described elsewhere [15].

### Library preparation and sequencing

DNA libraries were generated from the 24 extracted DNA samples with the Nextera XT DNA library preparation kit (Illumina Inc.) according to the manufacturer’s instructions. The samples were sequenced in one lane, on a Hiseq 4000, 150 bp pair-end with dual indexing at BGI, Copenhagen.

### Recovery and assessment of metagenomic assembled genomes

Trimmomatic v0.22 [65] was used to remove adapters and trim the reads (threshold quality = 15; minimum length = 40). Quality control was carried out using FastQC (Babraham Bioinformatics (http://www.bioinformatics.babraham.ac.uk/projects/fastqc/)). High-quality reads from each sample were co-assembled into scaffolds using IDBA-UD [66] with the options --pre_correction--min_contig 1000. In addition, 24 single-sample assemblies were performed following the same procedure. Metagenomic binning and refinement approach was conducted as previously described [67], and the overall workflow of the recovery of metagenomic assembled genomes is visualized in Figure S7.

In addition, metagenomic binning from several metagenomes downloaded from public databases (Table S1) was carried out using the binning, refinement and reassembly modules of metaWRAP [68]. Trimming and quality control of the raw reads as well as *de novo* assemblies of these metagenomes were carried out as describe above.

### Database construction

Fifty-five *Nitrospira* genomes recovered as part of this study and publicly available *Nitrospira* genomes were included in the genome dataset. Completeness and contamination rates of the all final population bins were assessed using CheckM [69]. A final set of 205 MAGs with completeness ≥ 70% and contamination ≤ 5% were included in the database.

For the metagenomic datasets, raw reads from 788 metagenomes downloaded (on January 2022) from NCBI [70] and MG-RAST [71] were included in the analysis. These metagenomes were selected as follows: The NCBI Sequence Read Archives (SRA) (sequencing type “whole genome sequencing”, “HiSeq”, and environmental package: “air”, “aquifer”, “biofilm”, “biofilter”, “coral reef”, “estuary”, “freshwater”, “groundwater”, “hot spring” “marine”, “metagenome”, “permafrost”, “rhizosphere”, “rice paddy”, “sand”, “sediment”, “soil”, “sponge” “urban”, “wastewater”, “wetland”) were queried with *amoA* and *nxrB* sequences from several *Nitrospira* spp. to identify datasets most likely to contain sequences associated with *Nitrospira* spp. Metagenomic datasets identified as possessing *Nitrospira* spp.-associated sequences were included in the metagenomic dataset. In addition, MG-RAST metagenomes not present in NCBI SRA, and with more than 2000 reads taxonomically annotated as *Nitrospira* were included in the metagenomic dataset. No more than six samples from the same study were included in the final metagenomic dataset used in this study to avoid their overrepresentation. Trimming and quality control of the raw reads as well as de novo assemblies were carried out as described above.

### Species abundance estimation

A 96% average nucleotide identity (ANI) cutoff was used to cluster genomes into groups of species as we identified a discontinuity in pairwise ANI for the 205 *Nitrospira* MAGs (Figure S8). Therefore, the *Nitrospira* genomes were dereplicated using dRep with the secondary clustering threshold set at 96% gANI. Among the genomes classified as belonging to the same species, the one with highest quality was chosen as representative for that species. The species abundance of each representative genome across the studied metagenomes was assessed using MIDAS [31]. Briefly, MIDAS uses reads mapped to 15 universal single-copy gene families (with ability to accurately recruit metagenomic reads to the correct species [31]) to estimate the abundance and coverage of bacterial species from a shotgun metagenome. We used the 132 *Nitrospira* species to build the database of universal-single-copy genes. Metagenomes were considered to contain *Nitrospira* spp. if at least eight reads mapped against the *Nitrospira* species present in our database of 15 universal single-copy genes. For richness calculation, the number of reads in each metagenome was normalized to 5 million and a *Nitrospira* species was considered to be present in a metagenome if at least an average (from 3 independent normalisations) of eight reads were mapped (as that would be above 50% of the 15 universal-single copy genes used by MIDAS). For the co-occurrence analysis, only *Nitrospira* species with at least one read per million (RPM) mapped on at least six metagenomes (above 1% of the metagenomes where *Nitrospira* spp. were detected) were considered.

### Comparative genome analysis

Two hundred five *Nitrospira* MAGs were included in the comparative genomic analysis. Gene calling was performed using Prodigal v.2.63 [72]. Annotation was conducted in RAST [73] and protein functional assignments of genes of interest were confirmed using blastp [74]. Pangenomic analysis was executed using the meta-pangenomic workflow of Anvi’o (v. 7.1) [30] with default parameters with the exception –maxbit = 0.3 (as descried in Palomo et al. [7]). Briefly, blastp was used to calculate similarities between all proteins in each genome. Weak matches between two protein sequences were eliminated using maxbit heuristic [75]. Finally, the Markov Cluster Algorithm [76] was used to generate gene clusters (GCs) from protein similarity search results. GCs were considered part of the core *Nitrospira* genome when present in at least 80% of the genomes. GCs were considered enriched in comammox *Nitrospira* when present in more than 60% of the comammox genomes (at least 40% in each clade) and absent in more than 90% of the non comammox genomes. GCs were classified as clade-specific (clade A, clade B, clade A1, and clade A2) if present in at least 55% of the clade-type genomes and absent in the rest of the *Nitrospira* genomes (Table S3).

### Phylogenetic analysis and gene synteny

Phylogenetic analyses of *Nitrospira* genomes were conducted with the GTDB-Tk v.0.1.3 tool [77] using the *de novo* workflow with a set of 120 translated universal single-copy genes and the genome taxonomy database (GTDB) [78]. Concatenated alignments were used to construct a maximum likelihood tree using RAxML v. 8.2.11 [79] with 200 rapid bootstraps (determined using the autoMRE option) and the LG likelihood model of amino acid substitution with gamma distributed rates and fraction of invariant sites (-m PROTGAMMAILGF; all substitution models were selected using ProtTest v. 3.4.2 [80]). The tree was rooted using two *Leptospirillum* species as outgroup. The online web tool from the Interactive Tree of Life (iTol) [81] was used for visualization. Predicted AmoA and HaoA amino-acid sequences were independently aligned with reference sequences using MUSCLE [82]. These alignments were used to construct maximum likelihood trees using RAxML v. 8.2.11 with 900 and 300 rapid bootstraps, respectively (determined using the autoMRE option). For AmoA, the tree was built using the LG model plus gamma distribution of rates across sites considering the amino acid frequencies of the data set evolution (-m PROTGAMMALGF), while for HaoA the tree was constructed using LG model with an estimation of invariable sites and gamma distribution (-m PROTGAMMALGF). Both trees were rooted using beta- and gammaproteobacterial AOB species as outgroup. The rooted trees were visualized using the online web tool from the Interactive Tree of Life (iTol) [81].

Gene arrangement of ammonia oxidation related genes was visualized using the R package genoPlotR [83].

### Statistical analyses

All statistical tests were performed using R v3.4.4 [84]. Due to the compositional nature of sequencing data [85], for all statistical analyses, species abundances were analyzed as follows: zeros were replaced with an estimate value using the count zero multiplicative approach with the zCompositions R package [86], and data were further centered log-ratio transformed. Statistical significance of the mean richness and abundance of *Nitrospira* species in the different habitats were assessed using Kruskal–Wallis ANOVA followed by Dunn’s test with the Holm-Bonferroni correction. Statistical significance of the mean richness and abundance between canonical and comammox *Nitrospira* species were evaluated using two-sided Mann-Whitney-Wilcoxon test.

Principal components analysis (PCA) was performed in R package factoextra [87]. Proportionality between abundances of the species across the 598 metagenomes were calculated using the propr R package [88] (with the options metric = “rho”, ivar = “clr”), which takes the compositional nature of the data into account, and visualized using the corrplot R package [89]. A Mantel test was used to investigate correlations between a matrix containing habitat similarity between species (as measured by the correlation between the abundances of *Nitrospira* species across samples) and a matrix of genome similarity (as measured by average nucleotide identity or sequence similarity of a set of 120 translated universal single-copy genes between pair of genomes) or amino acid sequence similarities of key nitrification enzymes (AmoA, HaoA, and NxrB). The significance of the Mantel statistic was obtained after 99,999 permutations.

*Nitrospira* community dissimilarities were calculated using the Jaccard index. The correlation between the *Nitrospira* community dissimilarities and geographic distances was calculated using the Mantel test (significance obtained after 99,999 permutations).

## Supplementary Information

**Additional file 1: Figure S1.** Pairwise average amino acid identity (AAI) of pairs of *Nitrospira* species. The AAI was clustered using average linkage hierarchical clustering based on pairwise Euclidean distances. Colour of the genome’s name indicates the *Nitrospira* type (see Fig. 4). **Figure S2.** Frequency of *Nitrospira* species occurrence in the metagenomes (*n* = 598) where at least one *Nitrospira* species was detected. Black, grey and white colors denote 0.5, 5, and 15 RPM of each species in each metagenome, respectively. **Figure S3.** Relationship between the community similarity and the geographic distance for seven different habitats. The dissimilarities between pairs of communities are calculated using the Jaccard index from a presence/absence matrix of *Nitrospira* genomes: the value 0 means that the two communities are the same. The Mantel test was used to test the strength and significance of correlations (*r* denotes the Mantel statistic *r*). Blue line shows the linear regression with shadowed region indicating 95% confidence intervals for the slope. The table shows the correlation (Mantel statistic r) between the community similarity and the geographic distance when all samples were analysed and when samples within short distances were excluded. **Figure S4.** Heat map analysis of *Nitrospira* species abundances across 598 metagenomes. Dendrograms are built based on Euclidean distance. Rows represent individual metagenomes and columns represent unique Nitrospira species. Colour intensity indicates center-log ratio transformed abundance. **Figure S5.** Correlogram showing the proportionality (ϼ) between the abundance of pairs of *Nitrospira* species across 598 metagenomes. Colour indicates whether the proportionality is positive (purple) or negative (brown). Size and darkness of the circles indicate the strength of the proportionality, with stronger proportionalities being larger and darker than weaker ones. A cut-off |ϼ| > 0.15 was chosen as it resulted in FDR < 0.001. Colour of the Nitrospira species indicates the type (see Fig. 4). **Figure S6.** Unique shared synteny between clade A2 and some of the clade B genomes in the hydroxylamine reductase genomic region (yellow arrows; duplicated NirK (CuNIR) in wine colour). Schematic of the ammonia oxidation pathway genomic region in comammox *Nitrospira* clade A1, clade A2, and clade B genomes. Functions of the encoded proteins are represented by colour. Single diagonal line designates a break due to contig fragmentation. Arrows represent genes, arrow direction denotes the orientation of the coding strand, and arrow lengths are proportional to the gene lengths. **Figure S7.** The implemented workflow for the MAGs recovery from 12 Danish groundwater-fed rapid sand filters. The final genome quality improvement performed with MeDuSa was only applied on the 18 *Nitrospira* MAGs. The numbers in the binning algorithm boxes indicate the minimum contig size considered for the binning step. For MetaBAT, SupB20 and SPpB20 indicate “superspecific” and “verysensitive” modes, respectively. **Figure S8.** Genetic discontinuity observed using 205 *Nitrospira* genomes. The scatter plot shows the pairwise MASHbased ANI (y axis) and ANIn (x axis) values among Nitrospira genomes. Only values in the 90–100% range are showed. Histogram plots show the distribution of MASH-based ANI and ANIn values among the *Nitrospira* genomes. A genetic discontinuum among the *Nitrospira* genomes is identified at around 96% for both metrics. **Supplementary Table S1.** List of publicly available metagenomes used to retrieve metagenome-assembled *Nitrospira* genomes. (provided as an Excel table). **Supplementary Table S2.** Characteristics of *Nitrospira* genomes. (provided as an Excel table). **Supplementary Table S3.** List of gene clusters of the *Nitrospira* pangenome. (provided as an Excel table). **Supplementary Table S4.** Characteristics of metagenomes. (provided as an Excel table). **Supplementary Table S5.** Richness and abundance of 132 *Nitrospira* species across habitats.

## Data Availability

All raw sequence data and genome sequences retrieved from the Danish rapid sand filters have been deposited at NCBI under the project PRJNA384587. The rest of the retrieved draft genomes are available on figshare (10.6084/m9.figshare.7999448). The file containing the gene clusters sequences of the *Nitrospira* pangenome is available on figshare (10.6084/m9.figshare.7998641). The data that support the plots within this paper are available from corresponding author upon reasonable request.
