# Supplementary material for: Genomic profiling of Nitrospira species reveals ecological success of comammox Nitrospira
Source: Microbiome. 2022 Nov 30;10:204. doi: 10.1186/s40168-022-01411-y (PMC9714041; doi:10.1186/s40168-022-01411-y)
Supplement: Supplementary file 2 — Additional file 1: Figure S1. Pairwise average amino acid identity (AAI) of pairs of Nitrospira species. The AAI was clustered using average linkage hierarchical clustering based on pairwise Euclidean distances. Colour of the genome’s name indicates the Nitrospira type (see Fig. 4). Figure S2. Frequency of Nitrospira species occurrence in the metagenomes (n = 598) where at least one Nitrospira species was detected. Black, grey and white colors denote 0.5, 5, and 15 RPM of each species in each metagenome, respectively. Figure S3. Relationship between the community similarity and the geographic distance for seven different habitats. The dissimilarities between pairs of communities are calculated using the Jaccard index from a presence/absence matrix of Nitrospira genomes: the value 0 means that the two communities are the same. The Mantel test was used to test the strength and significance of correlations (r denotes the Mantel statistic r). Blue line shows the linear regression with shadowed region indicating 95% confidence intervals for the slope. The table shows the correlation (Mantel statistic r) between the community similarity and the geographic distance when all samples were analysed and when samples within short distances were excluded. Figure S4. Heat map analysis of Nitrospira species abundances across 598 metagenomes. Dendrograms are built based on Euclidean distance. Rows represent individual metagenomes and columns represent unique Nitrospira species. Colour intensity indicates center-log ratio transformed abundance. Figure S5. Correlogram showing the proportionality (ϼ) between the abundance of pairs of Nitrospira species across 598 metagenomes. Colour indicates whether the proportionality is positive (purple) or negative (brown). Size and darkness of the circles indicate the strength of the proportionality, with stronger proportionalities being larger and darker than weaker ones. A cut-off |ϼ| > 0.15 was chosen as it resulted in FDR < 0.001. Colour of t [file 40168_2022_1411_MOESM1_ESM.zip › MS_Distribution_Nitrospira_new_SI_final.pdf]

Supplementary Information

Genomic profiling of *Nitrospira* species reveals ecological success of comammox *Nitrospira*

Alejandro Palomo[1,2*], Arnaud Dechesne[1], Anders G. Pedersen[3] and Barth F. Smets[1]
[1]Department of Environmental and Resource Engineering, Technical University of Denmark, Kgs Lyngby, Denmark
[2]School of Environmental Science and Engineering, Southern University of Science and Technology, Shenzhen, China
[3]Section for Bioinformatics, Department of Health Technology, Technical University of Denmark, Copenhagen, Denmark

Alejandro Palomo: alejandro@sustech.edu.cn *Corresponding author

Supplementary Information includes:
Figs. S1 to S8
References for SI reference citations

Other supplementary materials for this manuscript include the following:
Datasets S1 to S5

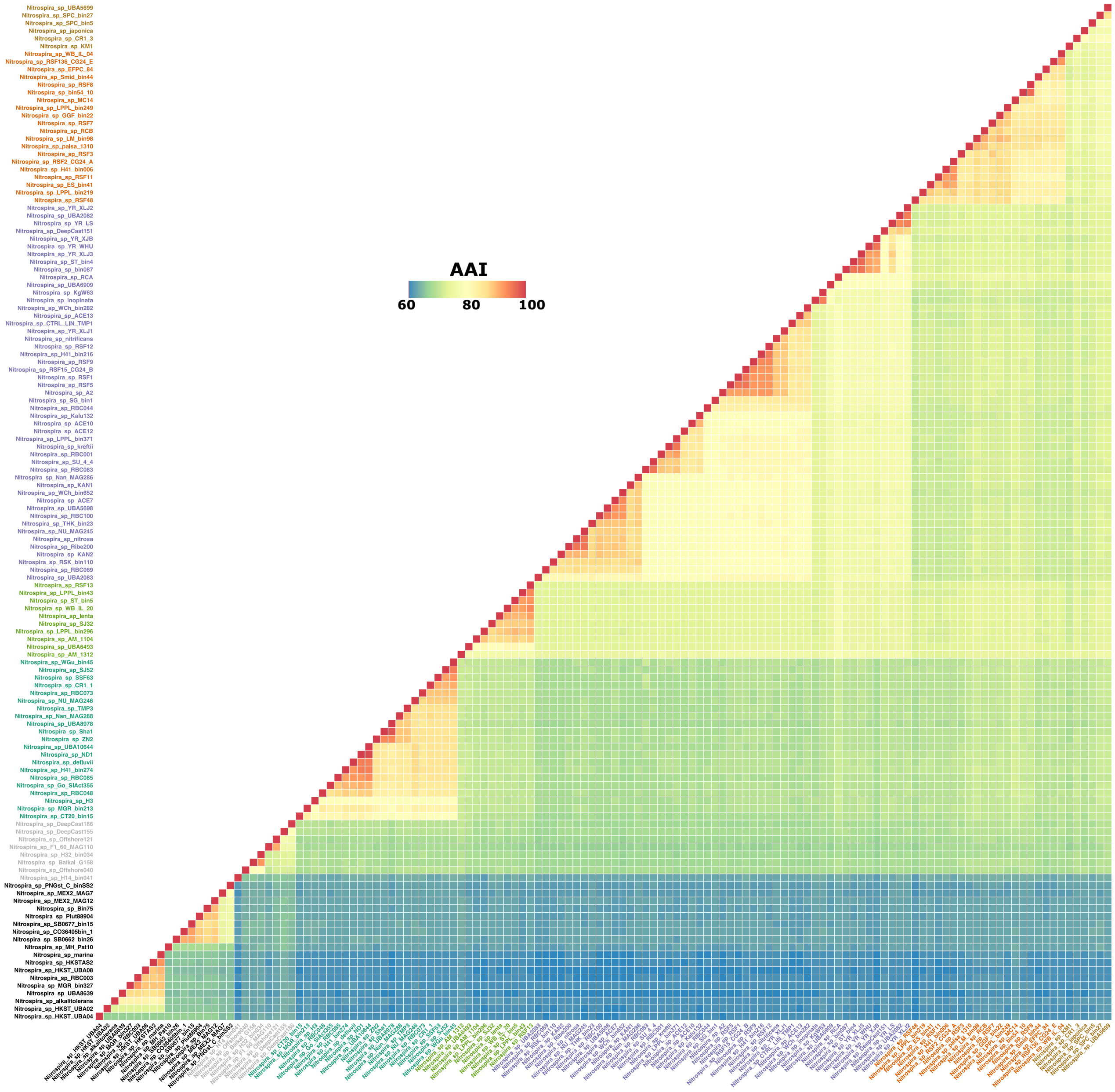

Fig. S1. Pairwise average amino acid identity (AAI) of pairs of *Nitrospira* species. The AAI was clustered using average linkage hierarchical clustering based on pairwise Euclidean distances. Colour of the genome's name indicates the *Nitrospira* type (see Fig. 4).

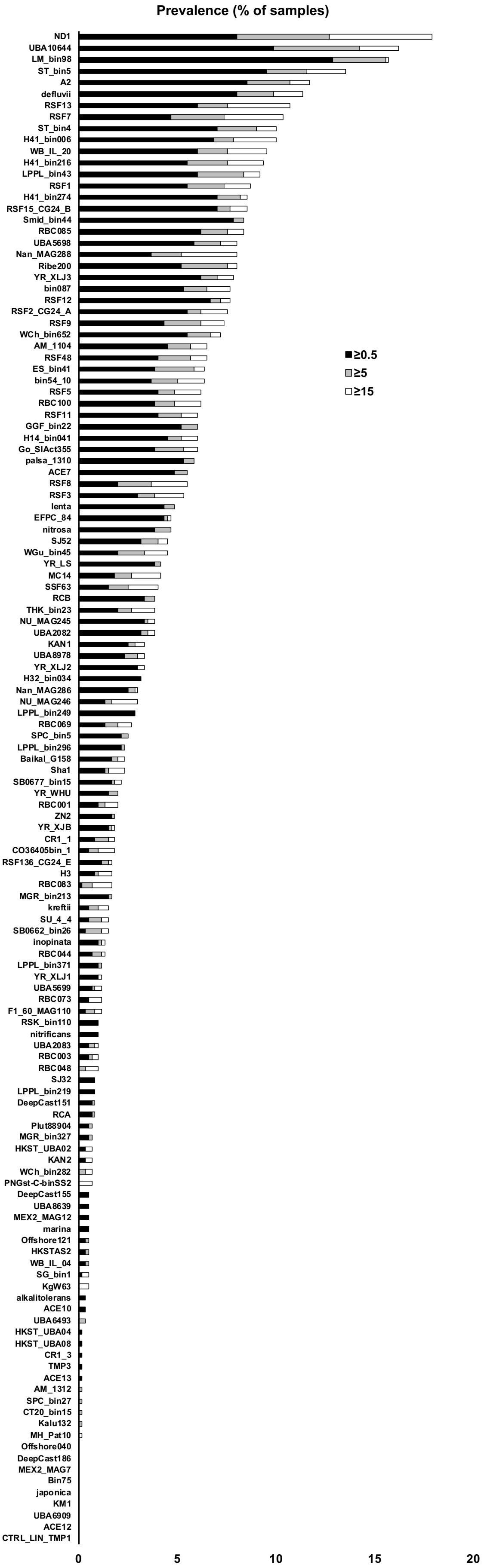

Fig. S2. Frequency of *Nitrospira* species occurrence in the metagenomes (n = 598) where at least one *Nitrospira* species was detected. Black, grey and white colours denote 0.5, 5, and 15 RPM of each species in each metagenome, respectively.

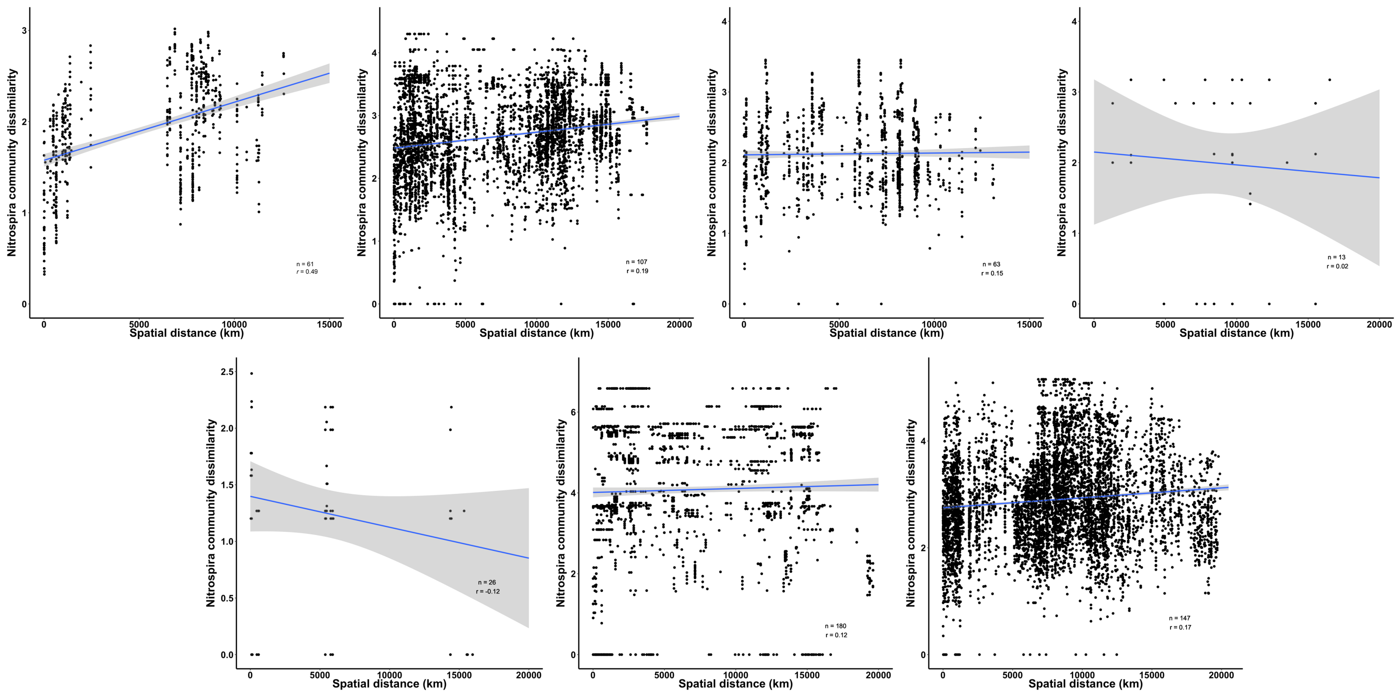

	DWTP	Freshwater	Groundwater	Hotspring	Marine	Soil	WWTP
All samples	0.49****	0.19****	0.15**	0.02	-0.12	0.12****	0.17****
Excluding 1 km	0.42****	0.14****	0.03	0.02	-0.25	0.08***	0.13***
Excluding 10 km	0.37****	0.12***	0.03	-0.05	-0.25	0.08***	0.13***
Excluding 100 km	0.34****	0.10**	-0.02	-0.05	-0.04	0.07**	0.12***
Excluding 1000 km	0.10	0.08*	-0.13	-0.05	-0.26	-0.07	0.05

* P < 0.01

** P < 0.001

*** P < 0.0001

**** P < 0.00001

Fig. S3. Relationship between the community similarity and the geographic distance for seven different habitats. The dissimilarities between pairs of communities are calculated using the Jaccard index from a presence/absence matrix of *Nitrospira* genomes: the value 0 means that the two communities are the same. The Mantel test was used to test the strength and significance of correlations (*r* denotes the Mantel statistic *r*). Blue line shows the linear regression with shadowed region indicating 95% confidence intervals for the slope. The table shows the correlation (Mantel statistic r) between the community similarity and the geographic distance when all samples were analysed and when samples within short distances were excluded.

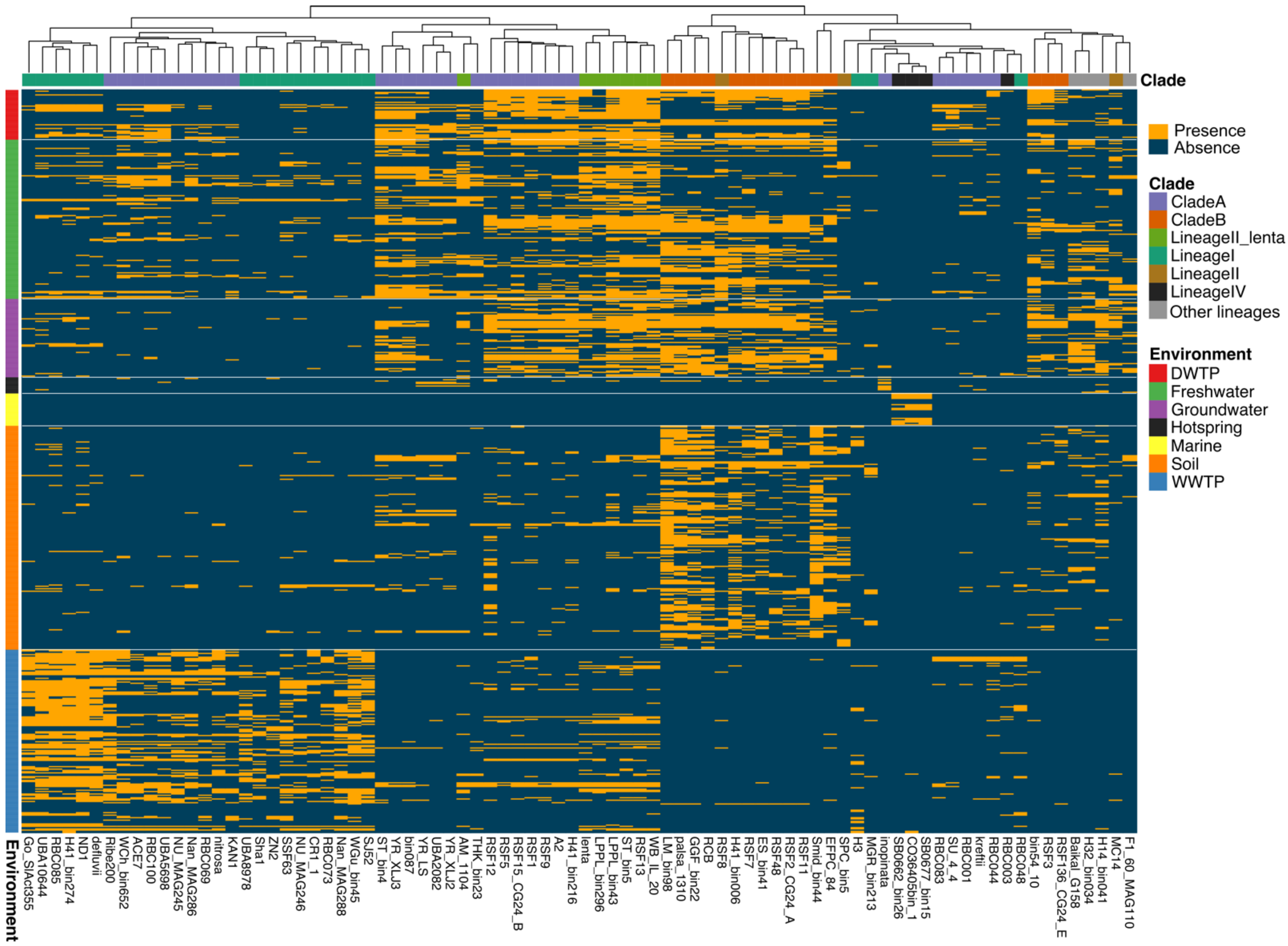

Fig. S4. Heat map analysis of *Nitrospira* species abundances across 598 metagenomes. Dendrograms are built based on Euclidean distance. Rows represent individual metagenomes and columns represent unique *Nitrospira* species. Colour intensity indicates centre-log ratio transformed abundance.

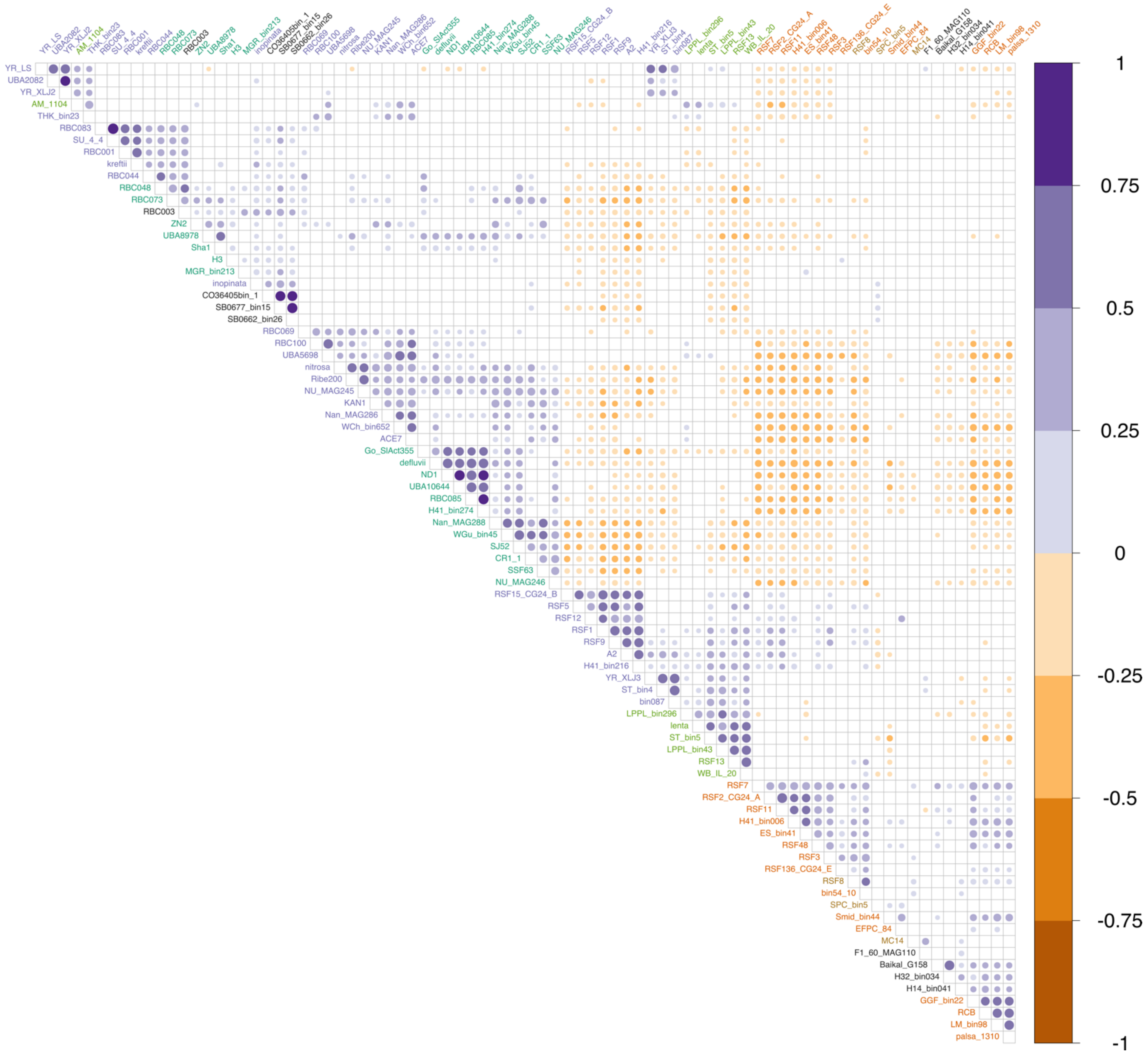

Fig. S5. Correlogram showing the proportionality (ρ) between the abundance of pairs of *Nitrospira* species across 598 metagenomes. Colour indicates whether the proportionality is positive (purple) or negative (brown). Size and darkness of the circles indicate the strength of the proportionality, with stronger proportionalities being larger and darker than weaker ones. A cut-off |ρ| > 0.15 was chosen as it resulted in FDR < 0.001. Colour of the *Nitrospira* species indicates the type (see Fig. 4).

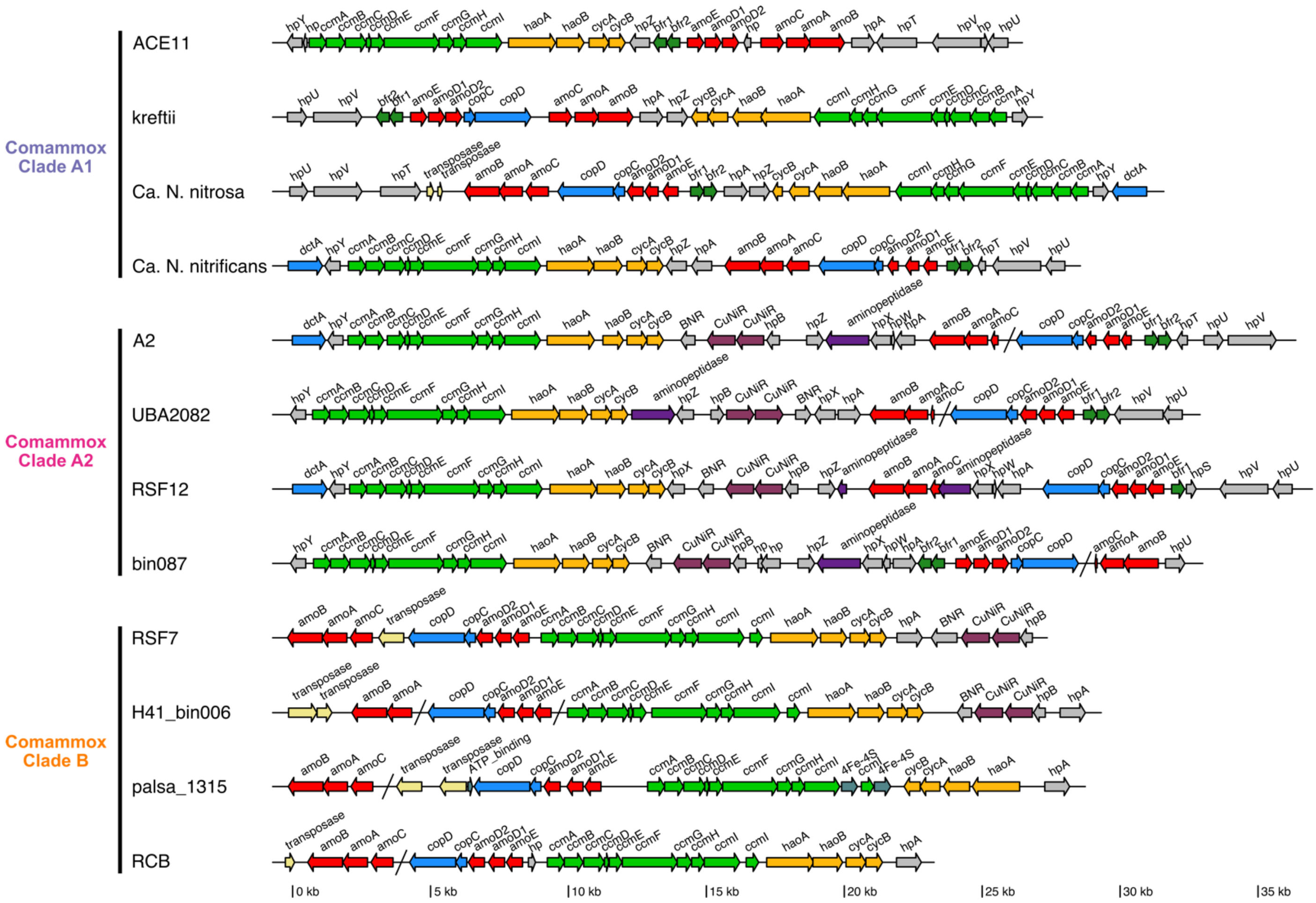

Fig. S6. Unique shared synteny between clade A2 and some of the clade B genomes in the hydroxylamine reductase genomic region (yellow arrows; duplicated NirK (CuNIR) in wine colour). Schematic of the ammonia oxidation pathway genomic region in comammox *Nitrospira* clade A1, clade A2, and clade B genomes. Functions of the encoded proteins are represented by colour. Single diagonal line designates a break due to contig fragmentation. Arrows represent genes, arrow direction denotes the orientation of the coding strand, and arrow lengths are proportional to the gene lengths.

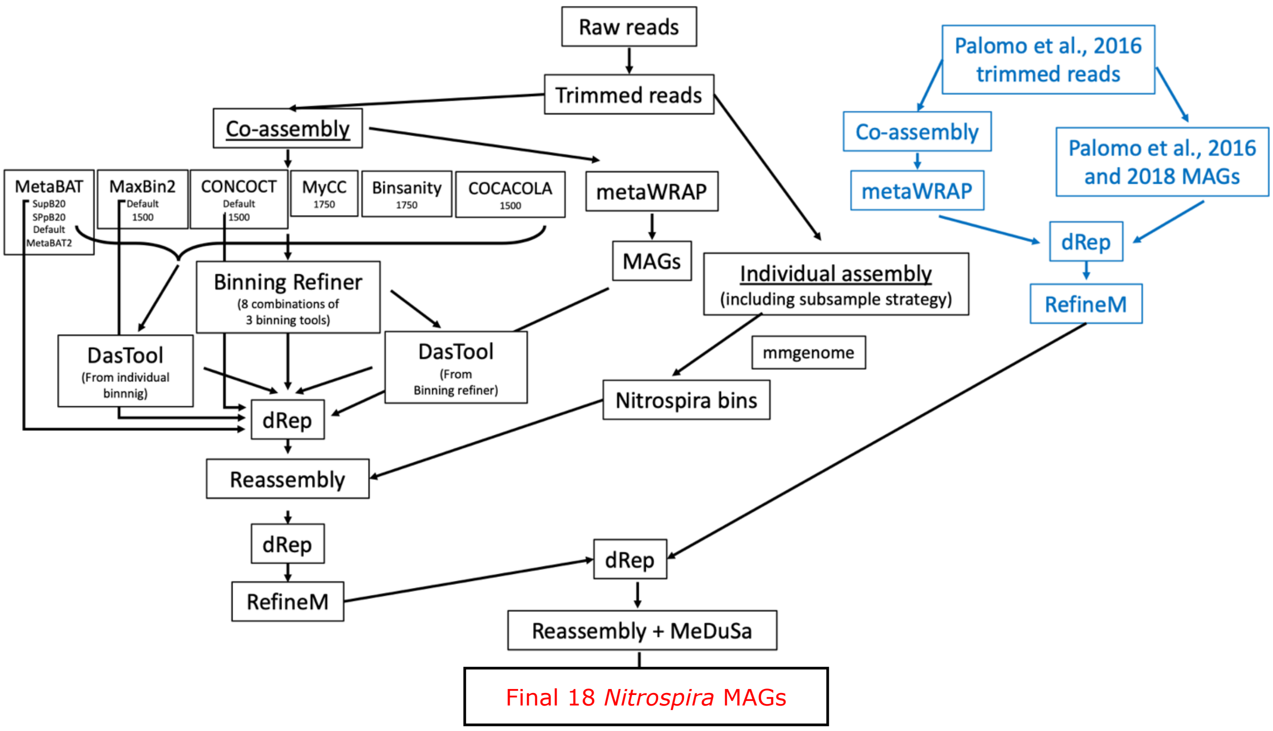

Fig. S7. The implemented workflow for the MAGs recovery from 12 Danish groundwater-fed rapid sand filters. The final genome quality improvement performed with MeDuSa was only applied on the 18 *Nitrospira* MAGs. The numbers in the binning algorithm boxes indicate the minimum contig size considered for the binning step. For MetaBAT, SupB20 and SPpB20 indicate "superspecific" and "verysensitive" modes, respectively.

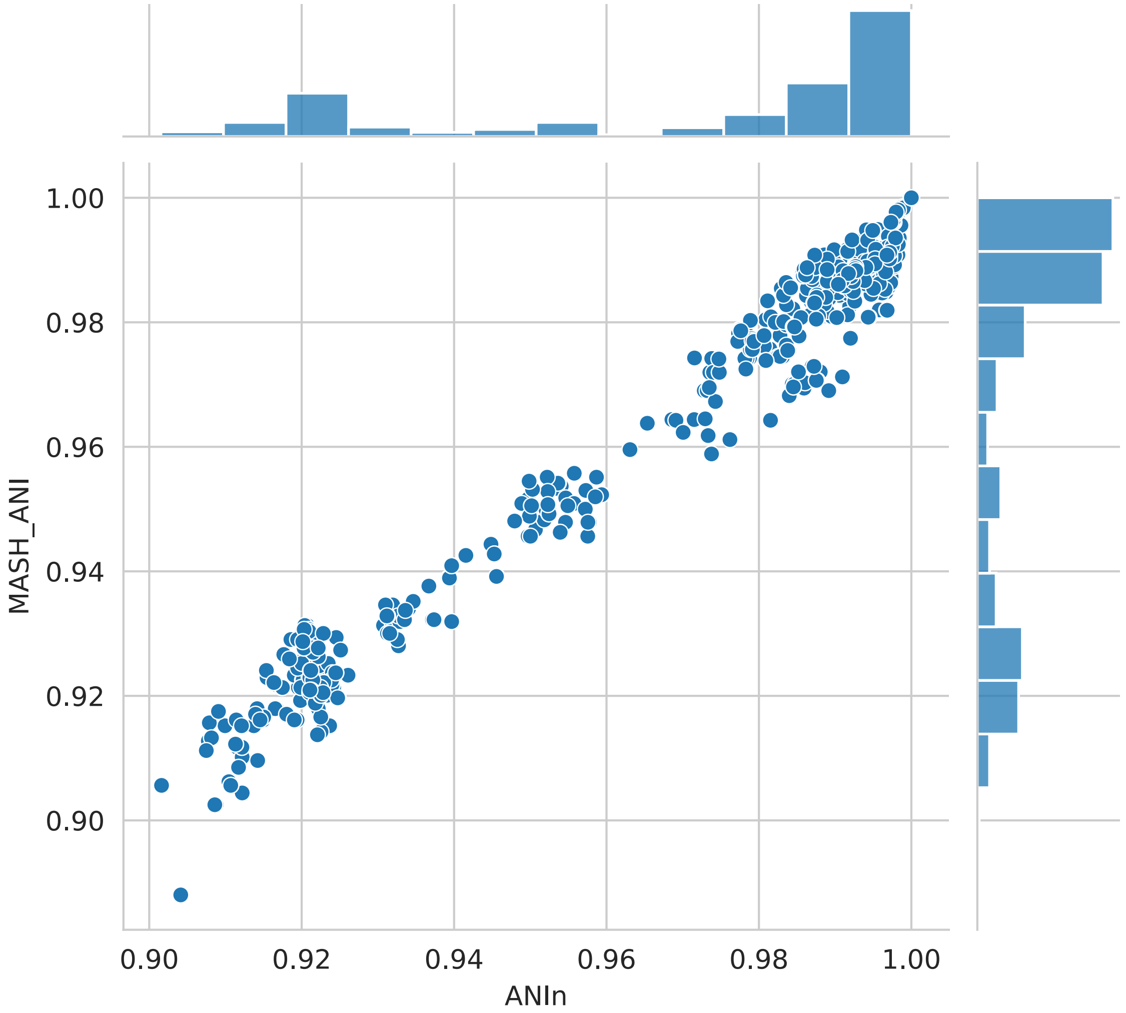

Fig. S8. Genetic discontinuity observed using 205 *Nitrospira* genomes. The scatter plot shows the pairwise MASH-based ANI (y axis) and ANIn (x axis) values among *Nitrospira* genomes. Only values in the 90–100% range are showed. Histogram plots show the distribution of MASH-based ANI and ANIn values among the *Nitrospira* genomes. A genetic discontinuum among the *Nitrospira* genomes is identified at around 96% for both metrics.

Supplementary Tables

Supplementary Table 1. List of publicly available metagenomes used to retrieve metagenome-assembled *Nitrospira* genomes.
(provided as an Excel table)

Supplementary Table 2. Characteristics of *Nitrospira* genomes.
(provided as an Excel table)

Supplementary Table 3. List of gene clusters of the *Nitrospira* pangenome.
(provided as an Excel table)

Supplementary Table 4. Characteristics of metagenomes.
(provided as an Excel table)

Supplementary Table 5. Richness and abundance of 132 *Nitrospira* species across habitats.

	**Richness**			**Abundance (number of mapped reads)**		
**Environment**	**95% ANI***	**75% ANI***	**75% ANI****	**95% ANI***	**75% ANI***	**75% ANI****
DWTP	10.0 ± 6.7 (n=39)	13.4 ± 8.2 (n=39)	24.4 ± 11.6 (n=40)	1245 ± 1702 (n=39)	1470 ± 1810 (n=39)	1434 ± 1801 (n=40)
Freshwater	2.9 ± 2.9 (n=98)	4.5 ± 4.6 (n=103)	11.8 ± 10.7 (n=134)	179 ± 245 (n=98)	253 ± 315 (n=103)	198 ± 293 (n=134)
Groundwater	5.3 ± 5.4 (n=39)	6.5 ± 6.9 (n=53)	12.1 ± 11.7 (n=73)	312 ± 512 (n=39)	422 ± 771 (n=53)	310 ± 680 (n=73)
Hotspring	1.4 ± 0.7 (n=8)	1.5 ± 0.7 (n=15)	6.1 ± 5.5 (n=22)	55 ± 49 (n=8)	64 ± 39 (n=15)	47 ± 42 (n=22)
Marine	1.3 ± 0.6 (n=23)	2.1 ± 1.6 (n=28)	3.1 ± 2.6 (n=34)	162 ± 264 (n=23)	182 ± 242 (n=28)	151 ± 230 (n=34)
Soil	1.1 ± 0.4 (n=43)	1.6 ± 1.16 (n=79)	5.5 ± 3.2 (n=198)	23 ± 14 (n=43)	52 ± 27 (n=79)	32 ± 24 (n=198)
WWTP	4.1 ± 3.5 (n=122)	4.8 ± 3.9 (n=127)	9.5 ± 7.7 (n=156)	217 ± 295 (n=122)	249 ± 316 (n=127)	204 ± 300 (n=156)

Each metagenome was normalised to 5 million reads and a *Nitrospira* MAG was considered to be present in a metagenome if at least 2** or 8* reads were mapped
